# Perceived likelihood of becoming pregnant and contraceptive use: Findings from population-based surveys in Côte d'Ivoire, Nigeria, and Rajasthan, India

**DOI:** 10.1016/j.contraception.2021.02.002

**Published:** 2021-06

**Authors:** Suzanne O. Bell, Alison Gemmill

**Affiliations:** Department of Population, Family and Reproductive Health, Johns Hopkins Bloomberg School of Public Health, Baltimore, MD, United States

**Keywords:** Contraceptive use, Low-resource settings, India, Perceived pregnancy risk, Sub-Saharan Africa, Survey research

## Abstract

**Objective:**

Advancing reproductive autonomy requires targeted strategies and interventions that address barriers to contraceptive use. The primary objective of this study is to investigate whether perceptions of low pregnancy likelihood are associated with lower likelihood of using contraception among presumably fecund, sexually active women.

**Study design:**

We used population-based survey data of reproductive age women at risk of pregnancy collected in 2018 from Côte d'Ivoire (N = 1447), Nigeria (N = 4110), and Rajasthan, India (N = 1994). To assess one's perceived biological likelihood of pregnancy, we used 2 measures: likelihood following a single act of sex without contraception and likelihood following 1 year of regular sex without contraception. Response options included: definitely yes, maybe yes, maybe no, definitely no, and do not know. We conducted multivariable logistic regression to assess the relationship between each perception measure with odds of contraceptive use separately by country.

**Results:**

Perceived chance of definitely or maybe becoming pregnant after one act of sex without contraception ranged from 54.0% to 55.0% in Nigeria and Rajasthan to 80.0% in Côte d'Ivoire, while it was higher for regular sex without contraception (76.0%–85.1%). Multivariable results indicate that perceptions of pregnancy likelihood were associated with contraceptive use among presumably fecund women, with a stronger relationship observed in relation to cumulative likelihood (odds ratio 0.1–0.6) than likelihood after one act (odds ratio 0.4–0.8) and a dose-response pattern by strength of perceived chance.

**Conclusions:**

Results indicate that women's use of contraception in low-resource settings is associated with their perceived likelihood of becoming pregnant after unprotected sex.

**Implications:**

Findings suggest that understanding women's perceived likelihood of pregnancy may aid in the development of interventions to help women achieve their reproductive goals.

## 1 Introduction

Ensuring that individuals have the means to achieve their fertility desires, including avoiding pregnancies they do not wish to have, requires targeted strategies and interventions that address barriers to contraceptive use [1]. Although prior studies have identified commonly cited reasons for not using contraception—such as concerns regarding health risks or side effects [2]—much less research has focused on how women's^1^ perceived likelihood of becoming pregnant interact with their contraceptive decision-making, especially in low-resource contexts. In particular, women may forgo contraception if they perceive that their chances of becoming pregnant are low. These pregnancy likelihood perceptions, however, are not always accurate, and may confer a false sense of protection among women wishing to avoid pregnancy [3,4].

Perceptions of pregnancy likelihood have not been studied extensively, and we have limited descriptive knowledge of potential variation in such perceptions both within and across populations. Nevertheless, a growing body of research, mostly from the United States, highlights how pregnancy likelihood perceptions might impact contraceptive use behaviors among women at risk of unintended pregnancy. For example, Gemmill [3] finds that low perceived fecundity, a measure of one's ability to become pregnant and have a live birth, is associated with nonuse of contraception even after controlling for self-reported difficulties becoming pregnant. Other United States-based studies have also demonstrated associations between low perceived susceptibility to pregnancy and intercourse without contraception [5,6], as well as use of less effective contraceptive methods [6]. Measures of pregnancy likelihood perceptions pertaining to a single act of intercourse are also associated with contraceptive behaviors. Biggs et al. [5], in their study of family planning clients, found that women were more likely to use contraception if they overestimated the likelihood of pregnancy from one act of intercourse without contraception.

There are few quantitative investigations of pregnancy likelihood perceptions in low- and middle-income settings, even though the topic of perceived likelihood of becoming pregnant and its interaction with contraceptive use has emerged in qualitative work [7,8]. Moreover, perceptions of infertility or problems becoming pregnant may be more common in low-resource settings, where the burden of infertility is disproportionately high [9], [10], [11] and concerns around reproductive potential are tied to cultural expectations of motherhood [9,[12], [13], [14]]. Knowledge about pregnancy likelihood may also be low in populations with lower reproductive health literacy [15]. A recent study from Polis et al. [16] begins to address this knowledge gap. Their study of young people in a region of Malawi found that 8% of women (and 20% of nulliparous women) believed they would have difficulty becoming pregnant when they wanted to [16]. Women who reported not using contraception had twice the odds of perceived potential infertility compared with women using modern or traditional contraceptive methods.

The primary objective of this study is to investigate whether perceptions of low pregnancy likelihood are associated with lower likelihood of using contraception among presumably fecund, sexually active women. The current study adds to the literature by examining this relationship across three diverse, low-income contexts; using two measures of pregnancy likelihood perceptions (i.e., single act and cumulative likelihood) that to our knowledge have not been used in these settings, and; documenting the demographic and reproductive characteristics associated with women's perceptions of pregnancy likelihood. We use data from Côte d'Ivoire, Nigeria, and Rajasthan, India, where recent data collection activities afforded the opportunity to add questions to assess perceptions of pregnancy likelihood. In Rajasthan, similar to India more broadly, women tend to finish childbearing early and then receive a tubal ligation, which is the most common contraceptive method. Conversely, short acting, nonpermanent methods are more frequently used in West African countries and longer birth spacing is more common. Thus, our 3-study settings provide 2 distinct reproductive contexts in which to examine our research question.

## 2 Methods

### 2.1 Data

Data for this study come from population-based surveys of reproductive age women (15–49 years old) conducted by Performance Monitoring for Action (PMA) in Côte d'Ivoire, Nigeria, and Rajasthan, India. PMA employs a stratified multistage cluster sampling design using probability proportional to size sampling of clusters (each comprised of approximately 200 households) to produce nationally and/or subnationally representative household and female samples to track key family planning indicators. Interviewers map and list all households in selected clusters and invite 35 (40 in Lagos, Nigeria) randomly selected households to participate in the survey. The sampling methodology is described in greater detail elsewhere [17]. Trained female interviewers conducted face-to-face interviews with selected households and all resident women aged 15 to 49. Interviewers obtained informed consent from women in accordance with local ethical committee approvals. Surveys typically lasted less than 45 minutes and covered women's socioeconomic characteristics, their reproductive history, their knowledge of and experience using contraception, and abortion. In Nigeria, interviewers conducted surveys using the English questionnaire or the translated versions in Hausa, Igbo, Yoruba, and Pidgin, while in Côte d'Ivoire and Rajasthan surveys were conducted in French and Hindi, respectively. The Comite National D’ Etique de la Recherche (CNER) in Côte d'Ivoire, the National Health Research Ethics Committee of Nigeria, and the Indian Institute of Health Management Research (IIHMR) Institutional Review Board for Protection of Human Subjects in Rajasthan provided ethical approval for the study protocol, as did the Johns Hopkins Bloomberg School of Public Health Institutional Review Board. Data collection occurred from July through August 2018 in Côte d'Ivoire, April through May 2018 in Nigeria, and April through June 2018 in Rajasthan.

### 2.2 Measurement

To assess women's perceived likelihood of becoming pregnant, we added 2 questions to the female questionnaire in the most recent survey rounds in each location. The English and translated language varied slightly across contexts, but sought to capture the intent behind the following 2 questions: “Depending on your body, if you have sex only once without using birth control, will you get pregnant?” and “Depending on your body, if you have sex regularly, say twice a week, for a year without using birth control, will you get pregnant?” “Depending on your body” was meant to capture one's personal biological likelihood of pregnancy, not a general woman's likelihood. The translation of this phrase into local languages varied but the meaning was similar. The response options included: definitely yes; maybe yes; maybe no; definitely no; and do not know. From these 2 questions, we created 2 separate measures of pregnancy likelihood—single act and cumulative—that retained all possible response options, including “do not know”, as individual categories. However, because only a small proportion of women (0.7–1.2%) provided a “do not know” response in Côte d'Ivoire and Rajasthan, pregnancy likelihood measures for these two settings exclude these women. This category of women who reported “do not know” was much larger in Nigeria (more than 10.0%), but we excluded these women from our analytic sample to maintain comparability across countries in our main analyses. The resulting main independent variables for single act and cumulative likelihood perceptions, therefore, included a 4-category variable in all 3 settings.

The dependent variable for this study was current contraceptive use, which captures reported use of any modern or traditional contraceptive methods by the woman or her partner at the time of the survey. Women who reported using any method were coded as 1, while those who reported no method were coded as 0.

Similar to other work that has examined pregnancy likelihood perceptions and contraceptive use, we view the underlying relationship as being best understood through the lens of the Health Belief Model [18], [19], [20]. As this was a secondary data analysis, we did not have the ability to model several factors included in prior conceptual frameworks, but we adjusted for available potential confounders based on existing literature [3,16,18,19,21]. Socioeconomic variables included measures of age (5-year age groups), education (none, primary, secondary, higher), marital status (currently married/cohabiting with a man, divorced/widowed, never married), wealth (a quintile based on household assets), and urban/rural residence (also state in Nigeria). Reproductive characteristics included parity (0, 1-2, 3-4, and 5 or more), recent sex (in the last month), and whether the woman wants a/another child in the future. We coded categorical variables as indicator variables.

### 2.3 Analysis

To begin, we restricted the analytic population to women who were at risk of unintended pregnancy in the absence of contraceptive use and were presumably fecund. In doing so, we excluded sterilized women or women whose partners had a vasectomy, women who self-identified as menopausal or had a hysterectomy, women who had not menstruated in the last 3 months, currently pregnant women, women who had never had sex, and women who wanted to get pregnant soon (within 1 to 2 months of the survey).

Among eligible women in each setting, we conducted univariate analyses to determine the overall distribution of perceived pregnancy likelihood after sex without contraception once and regularly over the course of a year, as well as women's sociodemographic and reproductive characteristics. We then conducted bivariate analyses to examine the relationship between covariates of interest and the independent variables. To assess statistical significance in these bivariate relationships, we used design-based F-tests. We conducted multivariable logistic regressions examining the independent relationship between perceived likelihood of becoming pregnant on contraceptive use adjusted for potential confounders identified *a priori*, including age, education, marital status, wealth quintile, residence, parity, whether the respondent had sex in the last month, and whether the respondent wanted a/another child, regardless of their significance in the bivariate analyses. We conducted analyses separately for single-act and cumulative measures of perceived pregnancy likelihood. We also stratified analyses by country.

To examine whether our findings were sensitive to certain assumptions or varied for some subpopulations, we conducted a number of sensitivity analyses. The relationship between pregnancy likelihood perceptions and contraceptive use may differ by age, as older women may correctly assume a lower probability of pregnancy due to age-related declines in fecundity. Women who intend to have children in the future might also engage in different contraceptive behaviors from those who intend to have no or no more children. Therefore, to assess the robustness of these findings, we separately tested for interactions by age (less than 35 years versus 35 years or older) and intentions for children (women who reported not wanting any/any more children in the future versus women who wanted a/another child in the future.) Second, to examine whether contraceptive method type modified the observed relationship, we compared use of long-acting reversible contraception, condoms, and other short acting nonbarrier contraceptives (pills, traditional methods, etc.) to no contraceptive use in separate multivariable models. Lastly, in Nigeria, we conducted a sensitivity analysis including the women who reported “do not know” to our two variables assessing perceived likelihood of pregnancy to observe whether findings were sensitive to the exclusion of this group. We weighted all analysis using the Taylor linearization method to account for the complex sampling design and clustering and conducted all analyses in Stata 15.1 [22].

## 3 Results

In Table 1 we present the descriptive characteristics of the sample. While total samples of interviewed women were 2738 in Côte d'Ivoire, 11,106 in Nigeria, and 5832 in Rajasthan, the analytic samples of presumably fecund, sexually active women were 1447, 4110, and 1994, respectively. The population in each setting was generally young, with most never attending school in Côte d'Ivoire and Rajasthan and most women attending at least some secondary school or higher in Nigeria. In Côte d'Ivoire and Nigeria approximately two-thirds of women were currently married or cohabiting compared to more than 90.0% in Rajasthan. A majority of women lived in urban areas in Côte d'Ivoire and Nigeria, whereas the population was primarily rural in Rajasthan. Among women in our sample, fertility was higher in Côte d'Ivoire and Nigeria, where approximately one quarter of women had 5 or more children and 36.0% and 43.5% were using contraception, respectively. In contrast, only 7.9% of women in Rajasthan had five or more children and almost half of women (47.6%) were using contraception. Only half of Rajasthani women had had sex in the month prior to the survey compared to approximately two-thirds of Nigerian and Ivoirian women. With regard to the one act of sex without contraception scenario, 53.9% of women in Nigeria indicated they would definitely or maybe become pregnant, compared to 55.0% in Rajasthan, and 80.0% in Côte d'Ivoire. In all 3 countries, more women thought they would definitely or maybe become pregnant when considering the scenario of regular sex without contraception for a year; 76.0%, 77.0%, and 85.1% in Nigeria, Rajasthan, and Côte d'Ivoire, respectively.

**Table 1:** Background characteristics among analytic sample of presumably fecund, sexually active Ivorian, Nigerian, and Rajasthani women age 15 to 49, 2018^a^

	Côte d'Ivoire (*N* = 1447)	Nigeria (*N* = 4110)	Rajasthan (*N* = 1994)
	*N* (%)	*N* (%)	*N* (%)
Age			
15–19	214 (15.5)	375 (8.9)	133 (5.9)
20–24	264 (17.7)	629 (15.5)	489 (24.3)
25–29	277 (18.5)	767 (19.2)	505 (25.2)
30–34	240 (16.9)	677 (15.9)	334 (17.0)
35–39	206 (14.6)	700 (17.3)	268 (13.7)
40–44	142 (9.4)	564 (13.8)	147 (8.3)
45–49	104 (7.4)	398 (9.4)	118 (5.6)
Education			
Never	685 (46.3)	763 (15.3)	670 (32.6)
Primary	374 (24.9)	720 (15.3)	531 (25.6)
Secondary	298 (22.2)	1824 (46.8)	323 (17.1)
Higher	90 (6.6)	803 (22.6)	470 (24.7)
Marital status			
Currently married/cohabiting	952 (65.3)	2980 (69.2)	1882 (94.8)
Divorced/widowed	84 (5.7)	300 (7.1)	78 (3.7)
Never married	411 (29.0)	829 (23.7)	34 (1.5)
Residence			
Rural	585 (40.9)	1953 (38.8)	1412 (59.6)
Urban	862 (59.1)	2157 (61.2)	582 (40.4)
Wealth			
Poorest	284 (20.5)	1027 (21.4)	312 (15.8)
Second poorest	296 (20.3)	893 (18.1)	311 (15.1)
Middle	263 (16.3)	748 (18.1)	384 (17.9)
Second wealthiest	295 (19.6)	730 (21.0)	471 (24.5)
Wealthiest	309 (23.3)	712 (21.4)	516 (26.8)
Parity			
0	249 (17.8)	836 (22.6)	257 (13.0)
1–2	465 (32.1)	986 (24.5)	1091 (54.6)
3–4	356 (24.4)	1140 (29.0)	486 (24.5)
5+	374 (25.7)	1143 (23.9)	160 (7.9)
Sex in last month	928 (63.8)	2933 (69.3)	1639 (47.6)
Wants a/another child	275 (18.8)	1328 (32.4)	1122 (57.3)
Current contraceptive use	520 (36.0)	1658 (43.5)	920 (47.6)
Perceived likelihood of becoming pregnant after sex without contraception once			
Definitely yes	590 (42.8)	1064 (24.5)	472 (23.7)
Maybe yes	559 (37.2)	1293 (29.5)	647 (31.3)
Maybe no	173 (10.8)	723 (19.3)	555 (27.3)
Definitely no	125 (9.1)	1030 (26.8)	320 (17.7)
Perceived likelihood of becoming pregnant after regular sex without contraception for 1 year			
Definitely yes	799 (56.7)	1708 (41.8)	829 (41.5)
Maybe yes	448 (28.4)	1435 (34.2)	738 (35.5)
Maybe no	133 (8.7)	476 (12.5)	278 (14.5)
Definitely no	67 (6.2)	491 (11.5)	149 (8.4)
Total	1447 (100.0)	4110 (100.0)	1994 (100.0)

a Percents are weighted to account for complex sampling design, Ns are unweighted.

Tables 2 and 3 display the bivariate relationships between each characteristic and both the single-act and cumulative pregnancy likelihood perception measures, respectively. We present row rather than column percentages to better facilitate comparisons across the three settings. Several factors were related to women's perception of short-term and long-term pregnancy likelihood following sex without contraception. Across all contexts, current contraceptive use was consistently associated with perceived likelihood of pregnancy after a single or regular exposure to sex without contraception, with more users indicating they would definitely become pregnant in either scenario compared to non-users (Tables 2 and 3). Several other covariates were also associated with perceptions of pregnancy likelihood, although relationships were not consistent across settings. Education was most consistently associated with perceptions of pregnancy likelihood in each context, with more educated women reporting a higher level of perceived pregnancy likelihood, although in Nigeria, highly educated women were just as likely to endorse “definitely yes” and “definitely no” for the single act measure. In Côte d'Ivoire and Rajasthan, greater wealth was associated with higher perceived likelihood of definitely becoming pregnant in relation to the single act measure, while this relationship was not as apparent in Rajasthan but was present in Côte d'Ivoire and Nigeria with regards to the cumulative measure. There was some indication that the youngest and oldest women rated their likelihood of becoming pregnant as lower, especially for the cumulative measure; only Nigeria showed a relationship with age with regard to the single act measure. The relationship between other characteristics and perceived likelihood of becoming pregnant did not reveal a consistent pattern across countries or exposure measures.

**Table 2:** Percent distribution of perceived likelihood of becoming pregnant after having sex without contraception *once*, overall and by background characteristics, among presumably fecund, sexually active women age 15 to 49 in Côte d'Ivoire, Nigeria, and Rajasthan^a^

	Côte d'Ivoire					Nigeria					Rajasthan				
	Def. yes	Maybe yes	Maybe no	Def. no	*p*-value	Def. yes	Maybe yes	Maybe no	Def. no	*p*-value	Def. yes	Maybe yes	Maybe no	Def. no	*p*-value
Age															
15–19	38.2	37.2	12.9	11.7	0.33	18.8	35.1	28.0	18.2	<0.01	24.5	27.6	27.8	20.1	0.42
20–24	40.3	40.8	10.2	8.7		23.6	31.6	20.3	24.5		24.4	33.6	26.6	15.4	
25–29	43.0	38.6	9.8	8.6		27.9	31.8	16.2	24.1		24.0	32.7	24.5	18.8	
30–34	41.9	40.1	11.3	6.7		28.1	27.7	17.3	26.9		25.4	32.7	26.1	15.8	
35–39	50.9	34.3	8.1	6.7		24.5	28.1	15.8	31.6		21.5	33.1	30.9	14.5	
40–44	47.9	29.7	13.4	8.9		21.2	29.1	19.1	30.6		20.1	26.8	28.7	24.4	
45–49	37.4	34.6	11.5	16.5		22.7	22.0	25.5	29.9		23.4	17.1	36.2	23.4	
Education															
Never	33.8	42.1	13.3	10.8	<0.01	14.3	37.2	23.6	24.9	0.01	18.5	31.7	29.8	20.1	0.08
Primary	49.9	33.7	7.8	8.6		24.3	27.9	19.2	28.6		21.2	33.1	27.5	18.2	
Secondary	49.4	34.5	8.3	7.9		25.7	28.8	17.8	27.8		27.8	26.4	26.3	19.5	
Higher	57.2	25.6	13.5	3.8		29.0	26.8	19.3	25.0		30.3	32.3	24.8	12.6	
Marital status															
Currently married/cohabiting	42.3	37.8	10.8	9.1	0.80	24.6	29.3	18.3	27.8	0.49	24.0	31.7	26.9	17.4	0.46
Divorced/widowed	51.0	32.3	11.5	5.2		25.5	28.1	18.3	28.1		18.1	25.1	31.0	25.8	
Never married	42.3	37.1	10.7	9.9		23.8	30.2	22.4	23.7		20.6	21.8	43.3	14.3	
Residence															
Rural	36.0	43.2	12.7	8.0	0.23	22.6	33.2	18.9	25.4	0.40	17.6	31.0	32.1	19.4	0.01
Urban	47.5	33.1	9.5	9.9		25.7	27.1	19.5	27.7		32.7	31.7	20.4	15.1	
Wealth															
Poorest	36.3	43.3	11.4	9.0	0.03	15.4	38.2	24.9	21.4	<0.01	17.1	26.1	31.2	25.6	0.06
Second poorest	34.8	44.8	10.7	9.8		26.6	27.3	16.4	29.6		17.4	30.8	34.8	17.0	
Middle	37.6	37.9	15.5	9.0		28.6	27.3	17.1	27.0		22.1	34.5	26.7	16.7	
Second wealthiest	43.7	40.3	6.8	9.2		25.7	25.6	20.1	28.6		23.3	35.5	25.9	15.3	
Wealthiest	58.4	22.2	10.6	8.7		26.9	28.2	17.0	27.9		32.5	28.6	22.7	16.2	
Parity															
0	45.7	30.8	12.1	11.4	0.22	21.3	31.7	23.1	23.8	0.03	24.4	26.6	28.7	20.4	0.28
1–2	43.8	39.1	10.1	7.0		25.1	29.8	19.1	25.9		24.7	33.5	24.6	17.3	
3–4	39.8	40.6	8.3	11.2		24.7	26.4	16.4	32.5		22.7	30.6	30.5	16.2	
5+	42.4	36.3	13.1	8.3		26.4	30.7	19.3	23.6		18.7	26.2	34.7	20.4	
Sex in last month															
No	45.0	35.8	9.7	9.5	0.59	26.0	28.3	17.7	28.0	0.42	20.3	25.5	33.3	20.9	0.05
Yes	41.6	38.1	11.5	8.9		23.8	30.0	19.9	26.3		24.4	32.5	26..1	17.0	
Wants a/another child															
No	41.7	38.4	10.7	9.3	0.31	23.1	31.0	19.5	26.3	0.17	25.1	31.6	25.8	17.5	0.68
Yes	47.7	32.4	11.6	8.4		27.2	26.2	18.8	27.8		22.6	31.1	28.5	17.8	
Current contraceptive use															
No	36.9	39.2	12.8	11.1	<0.01	19.2	30.0	20.7	30.1	<0.01	18.5	28.3	33.0	20.3	<0.01
Yes	53.2	33.7	7.4	5.7		31.3	28.7	17.4	22.6		29.4	34.6	21.2	14.8	
Total	42.8	37.2	10.8	9.1		24.5	29.5	19.3	26.8		23.7	31.3	27.3	17.7	

a Percents are weighted to account for complex sampling design.

**Table 3:** Percent distribution of perceived likelihood of becoming pregnant after having sex without contraception *regularly for one year*, overall and by background characteristics, among presumably fecund, sexually active women age 15 to 49 in Côte d'Ivoire, Nigeria, and Rajasthan^a^

	Côte d'Ivoire					Nigeria					Rajasthan				
	Def. yes	Maybe yes	Maybe no	Def. no	*p*-value	Def. yes	Maybe yes	Maybe no	Def. no	*p*-value	Def. yes	Maybe yes	Maybe no	Def. no	*p*-value
Age															
15–19	49.2	29.8	10.0	11.1	0.08	33.7	38.1	20.5	7.7	0.01	49.2	34.1	9.3	7.4	0.01
20–24	59.7	27.1	10.1	3.1		39.8	35.5	14.3	10.3		47.8	30.6	12.0	9.6	
25–29	61.3	27.6	7.3	3.9		46.7	33.2	10.9	9.2		43.4	34.7	16.0	5.9	
30–34	54.6	31.7	6.7	7.0		47.8	34.1	9.6	8.5		43.1	35.9	15.8	5.2	
35–39	64.1	27.3	6.6	1.9		40.7	33.0	10.0	16.4		30.5	47.5	14.1	7.9	
40–44	53.6	28.1	10.5	7.8		39.1	33.8	13.0	14.2		31.8	34.8	17.4	16.1	
45–49	47.9	26.0	12.9	13.2		38.3	33.5	13.9	14.3		34.3	32.6	17.7	15.4	
Education															
Never	49.7	33.5	11.0	5.8	0.01	22.3	42.9	21.8	13.0	<0.01	33.7	40.2	16.1	10.0	0.15
Primary	60.9	29.0	6.2	3.9		40.1	32.2	16.0	11.8		42.9	34.5	15.2	7.4	
Secondary	62.4	22.0	6.7	9.0		44.8	33.0	10.1	12.1		42.5	35.3	13.6	8.6	
Higher	71.0	12.4	9.2	7.4		50.0	32.3	8.6	9.1		49.7	30.5	12.4	7.4	
Marital status															
Currently married/cohabiting	54.3	30.6	9.0	6.0	0.23	40.4	34.1	12.7	12.9	0.09	41.9	35.3	14.6	8.2	0.45
Divorced/widowed	60.5	28.2	9.7	1.6		45.4	28.5	15.7	10.5		36.0	35.0	13.0	16.0	
Never married	61.3	23.5	7.8	7.4		44.9	36.2	11.0	7.9		30.3	49.9	14.6	5.2	
Residence															
Rural	44.9	39.1	10.8	5.2	0.01	37.3	36.0	15.4	11.2	0.22	41.4	34.9	15.7	8.1	0.87
Urban	64.8	21.1	7.3	6.8		44.6	33.0	10.6	11.7		41.8	36.4	12.9	9.0	
Wealth															
Poorest	46.2	37.7	10.0	6.2	<0.01	26.0	40.9	22.4	10.7	<0.01	32.6	32.8	19.8	14.8	0.06
Second poorest	43.6	41.5	10.6	4.4		42.2	31.9	13.6	12.3		34.9	39.6	19.9	5.6	
Middle	55.4	30.0	11.0	3.7		42.8	34.0	8.4	14.8		43.4	38.1	12.4	6.0	
Second wealthiest	66.0	21.0	6.4	6.5		47.3	32.9	8.8	11.0		47.7	34.6	12.0	5.7	
Wealthiest	70.4	14.1	6.4	9.1		50.9	30.9	8.7	9.5		43.6	33.8	12.2	10.4	
Parity															
0	57.8	22.2	8.8	11.1	0.03	40.8	38.6	12.6	8.1	0.14	41.7	34.0	13.9	10.5	0.68
1–2	61.7	25.0	8.5	4.8		42.9	33.8	11.8	11.5		43.6	34.2	14.4	7.8	
3–4	55.6	31.1	7.5	5.8		42.4	30.7	12.3	14.7		39.0	38.4	15.0	7.6	
5+	50.7	34.7	9.8	4.8		40.9	34.9	13.5	10.8		34.7	38.2	15.4	11.7	
Sex in last month															
No	57.3	28.9	7.8	6.0	0.84	45.1	34.5	9.2	11.2	0.02	37.6	36.0	15.8	10.6	0.42
Yes	56.3	28.2	9.3	6.2		40.3	34.1	13.9	11.7		42.4	35.4	14.3	8.0	
Wants a/another child															
No	56.4	28.4	8.5	6.8	0.41	40.3	35.6	13.3	10.8	0.06	44.8	32.9	13.5	8.8	0.24
Yes	58.1	28.7	9.9	3.3		44.9	31.2	10.8	13.1		39.1	37.5	15.3	8.2	
Current contraceptive use															
No	48.6	32.3	10.5	8.5	<0.01	31.6	38.5	15.5	14.4	<0.01	32.0	37.7	19.2	11.1	<0.01
Yes	71.0	21.6	5.5	1.9		55.0	28.7	8.5	7.8		51.9	33.1	9.5	5.4	
Total	56.7	28.4	8.7	6.2		41.8	34.2	12.5	11.5		41.5	35.5	14.5	8.4	

a Percents are weighted to account for complex sampling design.

As shown in Table 4, multivariable results among the full analytic sample suggested a consistent, dose-response relationship between level of perceived pregnancy likelihood and odds of current contraceptive use for both likelihood measures in nearly all models, even after adjusting for potential confounders. In Côte d'Ivoire, the adjusted model indicated that women who think they *may* become pregnant after one act of sex without contraception had 33% lower odds of currently using contraception compared to women who *definitely* think they will become pregnant (odds ratio [OR] 0.7), whereas women who thought they maybe would *not* become pregnant had even lower odds of currently using contraception (OR = 0.4), followed by women who thought they would *definitely not* become pregnant (OR = 0.4). The relationship became stronger when examining perceived likelihood of pregnancy after a year of regular sex without contraception, with adjusted ORs ranging from 0.1 to 0.6 for women in Côte d'Ivoire who thought they *may, may not*, or *definitely would not* become pregnant relative to women who thought they would *definitely* become pregnant. The results followed similar patterns in Nigeria, with analogous dose-response relationships; adjusted ORs ranged from 0.4 to 0.7 for one act of sex without contraception and 0.3 to 0.5 in the context of regular sex without contraception. The dose-response relationship was less pronounced in Rajasthan, with adjusted ORs ranging from 0.4 to 0.8 in relation to short-term likelihood and 0.3 to 0.5 in relation to long-term likelihood.

**Table 4:** Adjusted odds ratios for current use of contraception according to perceived likelihood of becoming pregnant after having sex without contraception once or regularly for one year among presumably fecund, sexually active women age 15–49 in Côte d'Ivoire, Nigeria, and Rajasthan

	Sex without contraception once			Sex without contraception regularly for one year		
	aOR^a^	95% CI		aOR^a^	95% CI	
Côte d'Ivoire (*N* = 1444)						
Definitely yes	1.00	–	–	1.00	–	–
Maybe yes	0.67	0.44	1.01	0.57	0.36	0.91
Maybe no	0.39	0.22	0.71	0.39	0.23	0.66
Definitely no	0.37	0.22	0.65	0.11	0.04	0.35
Nigeria (*N* = 4104)						
Definitely yes	1.00	–	–	1.00	–	–
Maybe yes	0.72	0.53	0.97	0.48	0.38	0.59
Maybe no	0.61	0.42	0.89	0.39	0.26	0.59
Definitely no	0.44	0.31	0.63	0.29	0.20	0.41
Rajasthan (*N* = 1960)						
Definitely yes	1.00	–	–	1.00	–	–
Maybe yes	0.76	0.49	1.16	0.54	0.37	0.77
Maybe no	0.43	0.28	0.67	0.29	0.17	0.51
Definitely no	0.54	0.31	0.92	0.33	0.18	0.60

a Adjusted for age, education, marital status, wealth quintile, residence (also state in Nigeria), parity, whether had sex in last month, and whether want a/another child.

Sensitivity analyses provided additional support for these relationships. With regard to interactions, there was little evidence of interaction by age or intentions for children (estimates not shown). However, there was one exception: older Rajasthani women (≥35) who thought they would *definitely not* become pregnant after a year of regular sex without contraception had the lowest adjusted odds of contraceptive use compared to other groups (estimates not shown). There were no significant interactions with age or sexual activity in Nigeria and Côte d'Ivoire, and no interactions with desire for children in any of the settings (estimates not shown). Exploring differences by method type, we found similar evidence of a dose response, most consistently with long-acting reversible contraceptions, where the ORs tended to be even further from the null, whereas patterns were similar for condom use and somewhat attenuated for other short-acting contraceptive methods. Lastly, the Nigeria-specific sensitivity analysis revealed that women who replied “do not know” to either perceived pregnancy likelihood question had the *lowest* likelihood of using contraception in the multivariable model, while the findings with regard to the other response options changed minimally.

## 4 Discussion

In three different low-resource settings, we find that perceptions of one's biological likelihood of pregnancy are independently associated with contraceptive use among women at risk of unintended pregnancy. Moreovert, we find the relationship demonstrates a clear dose-response pattern, suggesting that lower perceived likelihood of becoming pregnant is associated with lower odds of current contraceptive use. These findings also appear robust to model and subpopulation specifications and are consistent across diverse settings. Taken together, our results are in line with similar research in the United States that finds relationships between perceptions of pregnancy likelihood associated with a single act of sex without contraception or over a longer period of time and contraceptive use behaviors [3,5,6,23].

Notably, we find similar relationships between our 2 measures of pregnancy perceptions—single-act and cumulative—and contraceptive use. These similar patterns might be due to the possibility that both measures draw on the same latent construct (e.g., self-perceived fecundity) or reflect general notions of confidence or certainty. That said, at the population level, there does appear to be some distinction between the two measures, given that larger shares of women thought they would become pregnant after a longer period of exposure to sex without contraception. Thus, future research should evaluate the construct validity of different pregnancy likelihood perception measures to understand how respondents interpret such survey questions and formulate answers.

As demonstrated in Tables 2 and 3, we find striking variation across the three settings in women's self-perceptions of pregnancy likelihood, with Ivorian women reporting higher levels of perceived pregnancy likelihood than their Nigerian and Rajasthani counterparts. Some of these different patterns might be due to differences in population composition between the three settings. However, the relationship between pregnancy likelihood perceptions and women's demographic characteristics was not consistent across study settings. For example, while we might expect women over 40 to express similar levels of doubt in their fecundity across populations, we find that 47.9% to 53.6% of Ivorian women over 40 said that they would “definitely” become pregnant after a year of regular, unprotected sex compared with 38.3% to 39.1% of older Nigerian women and 31.8% to 34.3% of older Rajasthani women. These descriptive findings provide suggestive evidence that women may rely on culturally distinct schemas or cognitive structures when forming beliefs about their reproductive ability [24].

Based on responses to our measure of cumulative pregnancy likelihood perceptions (i.e., likelihood of getting pregnant after one year of regular sex without contraception), our findings also suggest that a nontrivial proportion of women in these contexts believe they may be infertile. Indeed, 11.5% of women in Nigeria, 8.4% of women in Rajasthan, India, and 6.2% of women in Côte d'Ivoire indicated they would definitely not become pregnant after a year of regular sex without contraception. Because women's perceptions of pregnancy likelihood have not been rigorously studied in low-resource settings, we have limited knowledge about the types of information that may inform these infertility beliefs. However, it may be the case that for many women, their subjective assessments of pregnancy likelihood closely align with reality [11]. It might also be the case, though, that some perceptions may be based on erroneous information [25], [26], [27] or inaccurate knowledge of the period in a woman's menstrual cycle when she is most at risk of pregnancy [15]. Perceptions might also be impacted by prior contraceptive use, as existing literature suggests that many women believe hormonal contraception harms future fertility [9,[12], [13], [14]].

A non-trivial proportion of presumably fecund women in Nigeria (10.3% and 11.1%), but not Côte d'Ivoire and Rajasthan, also provided a “do not know” response to the two pregnancy likelihood measures, suggesting they may not have considered this likelihood or had difficulty quantifying it when prompted. Notably, we observed in a sensitivity analysis that these women had the lowest odds of contraceptive use among the response categories, indicating that the typical practice of dropping “do not know” responses in analyses might systematically exclude the most vulnerable populations. The women who responded “do not know” in Nigeria were also half as likely to have had sex in the last month in multivariable analyses (estimates not shown), which may signal confusion regarding how to answer the question in the context of potentially less regular sexual activity.

This study has a number of strengths. We investigated this relationship in new settings using representative samples of reproductive age women. The data were contemporaneously collected and represent large, diverse samples in which to investigate this hypothesis. Adding questions on both perceived pregnancy likelihood associated with one act of sex without contraception and regular sex without contraception provided a more complete understanding of this relationship. Because other reproductive surveys conducted in low- and middle-resource countries (e.g., Demographic and Health Surveys) do not include these or similar measures, our study offers unique insights on this phenomenon in low-resource settings.

However, this research is not without limitations. Evidence suggests that standard survey approaches to measuring contraceptive use may fail to capture a substantial amount of traditional contraceptive use [7,28]. As such, many of the non-users in our study may in fact be protected through the use of withdrawal or rhythm method. Moreover, our measures of pregnancy likelihood have not been tested or validated. Hypothetical questions like the ones we used to measure these perceptions can be cognitively demanding and especially difficult to answer when translating into local languages or dialects that don't have a comparable tense. They are also open to different or misinterpretations, perhaps particularly so among women with limited sexual experience or who are using a long-acting reversible contraceptive method that may not be removed for several years. We note, however, that validated measures of pregnancy likelihood, especially in low-resource settings, are lacking, and we conducted cognitive testing and practice fieldwork in each setting to maximize correct interpretation. Another limitation is that we do not know if women's perceptions accurately reflect their actual underlying likelihood of pregnancy; for example, the data we use do not include measures of infertility or impaired fecundity. Finally, the cross-sectional nature of the data limits our interpretation of study findings. With cross-sectional data, we are unable to determine if lower perceptions of pregnancy likelihood lead to lower use of contraception or if prior instances of sex without contraception that did not result in a pregnancy led to lower perceived fecundability. Contraceptive nonuse could also be a result of previously experienced fertility issues. Additionally, we do not know if contraceptive nonuse among women holding low perceived likelihood of becoming pregnant ultimately leads to unintended pregnancy. Longitudinal data are needed to better understand the directionality and nature of these relationships.

Our findings have public health programmatic and policy implications. The results suggest that investigating and understanding women's pregnancy likelihood perceptions may aid in the development of interventions to help women avoid unintended pregnancy. While the actual probability of conception after a single act of sex without contraception is quite low (3%–10%) [29,30], many women in our study viewed their likelihood of becoming pregnant under these circumstances as higher (“definitely yes”). If one's expectations are inaccurate in this way, the experience of sex without contraception that does not result in pregnancy may give the false impression of an inability or difficulty to conceive. Increased access to quality sexual and reproductive health education that includes discussions of fertility awareness [31, 32] would provide women with a more accurate understanding of their short- and long-term pregnancy likelihoods so that women could more accurately gauge the level of pregnancy risk they are willing to accept at any point in time.

## Appendix Supplementary materials

Image, application 1

## Footnotes

1 We recognize that people of all genders have pregnancies. However, we use female identifiers in this paper as nearly all data on pregnancy in low-resource settings only capture people who identify as women.
